# Design of an ultrasound appointment system based on a patient-centered real-time dynamic resource allocation strategy

**DOI:** 10.3389/fpubh.2025.1496860

**Published:** 2025-02-24

**Authors:** Yong Zhang, Yan Luo, Li Qiu, Ying Zhu, Xiao Lu

**Affiliations:** ^1^Department of Medical Ultrasound, West China Hospital of Sichuan University, Chengdu, China; ^2^Department of Thoracic Surgery, West China Hospital of Sichuan University, Chengdu, China

**Keywords:** ultrasound appointment, patient-centered, dynamic resource, allocation strategy, real-time

## Abstract

**Introduction:**

The existing ultrasound appointment system faces multiple challenges, including a lack of diversity in its operation modes, sluggish efficiency, and limited flexibility. During emergency situations, such as disease outbreaks or severe disaster events, the demand for ultrasound examinations skyrockets, making it imperative to offer patients efficient and user-friendly ultrasound appointment services.

**Methods:**

This study introduces the application of a patient-centered real-time dynamic resource allocation strategy in an ultrasound appointment system. This strategy focuses on the demand of patients, builds a multichannel and multimode ultrasound appointment system, and opens sets of parameters related to ultrasound appointments, such as examination room attributes, workload adjustments, and mutually exclusive rules of medical orders. The system can display patient appointment data in real time and carry out statistical analysis, and medical resources can be flexibly configured according to the patient appointment situation to fully meet the needs of patients. Moreover, the system interconnects the appointment data with the registration system and the examination room examination list to further optimize the medical service process.

**Results:**

Data such as the proportion of appointment channels, appointment time, and the quality and efficiency of ultrasound examinations before and after the introduction of the appointment system were compared and analyzed. According to the statistics, the proportion of online bookings increased from 0% to 81.42%. The average appointment times of general ultrasound examination and specialist ultrasound examination were reduced by 90.7% and 78.86%, respectively. The appointment staff was saved by 4 people, the average waiting time of patients in the examination area was reduced from 42 min to 11 min, and the number of ultrasound examinations was increased by 11.5%, while the number of error reports was also significantly reduced.

**Discussion:**

The results show that the application of this strategy in an ultrasound appointment system is feasible and efficient. Patients can participate more in the entire process of ultrasound appointment and examination, obtain reliable ultrasound medical services faster and more efficiently, improve the diagnosis and treatment environment and order of the hospital, and optimize the medical service process.

## Introduction

With the rapid development and popularization of ultrasound medical technology, ultrasound medical examination and diagnosis have been applied throughout the body, including the abdomen, superficial surface, blood vessels, musculoskeletal system, and heart. According to statistics, ultrasound examination is an important part of modern imaging examination, and it accounts for more than 30% of all imaging examinations (1, 2). The number of ultrasound patients is large, but ultrasound medical resources are limited, so ultrasound is often conducted by appointment (3). The appointment system has achieved certain results in many fields, including students' appointments in classrooms, trucks, and hotels (4–6). However, the appointment in the medical system is relatively more complicated; in addition to issuing medical orders, payments, etc. (7), there are other restrictions on various examinations in the hospital, such as fasting, defecation, etc., and there may be restrictions and conflicts between different examinations, such as that abdominal ultrasound examination cannot be performed within 2 days after gastroenteroscopy (8). Although many medical systems have designed a variety of ultrasound appointment systems, most of them are still only for medical personnel, and patients need to make an appointment at the ultrasound appointment window after opening the examination application form and paying (9). This approach not only requires patients to queue several times but also requires multiple trips to and from the hospital, which is inefficient (10). When patients cannot attend appointments due to other matters, there is also a lack of effective modification channels, which may cause patients to miss appointments, further increasing the time of ultrasound appointments and wasting valuable medical resources (11, 12). To obtain a more suitable time when making an appointment, patients often arrive at the hospital many hours in advance to carry out an inspection as early as possible, wasting patients' time and causing a crowded hospital environment and difficulty maintaining order (13). When patients need more than one test, it is even less efficient and even ineffective because of inadequate preparation or conflicts between tests (14, 15). During disease outbreaks, like the COVID-19 pandemic, ultrasound appointment services can experience even greater delays (16, 17). In emergency scenarios such as floods, earthquakes, and other catastrophic events, the need for ultrasound examinations spikes significantly, hospitals are focusing on how to swiftly adapt to fluctuations in patient numbers, minimize fatalities, and uphold societal wellbeing (18, 19). Consequently, there's an urgent necessity to revise the current ultrasound appointment procedures and methodologies, as well as ultrasound resource allocation strategies. This is not only to address issues such as frequent patient visits, extended appointment times, and inconveniences, but also to enhance preparedness for a variety of diseases and disasters (20, 21). Many scholars have also conducted studies on the problems encountered in the ultrasound appointment system, including the cause of patient absenteeism, online appointment design, and cohort optimization (22, 23). These studies have achieved some results, but there are still some problems, such as insufficient channel smoothness, poor statistical and analytical utilization of data, inflexible configurations, and poor interactions between appointment data and other systems (24, 25).

In this study, we describe the application of a patient-centered real-time dynamic resource allocation strategy in an ultrasound appointment system. The strategy is built around the actual needs of patients and provides patients with online and offline multichannel and multimode ultrasound appointment selection. The system has a built-in ultrasound examination room, doctor's orders, a workload, mutually exclusive rules and other attributes. In accordance with the real-time appointment data of patients, the dynamic adjustment of ultrasound medical resources is carried out to meet the needs of patients. Moreover, the data of the appointment system are connected with the registration system, examination room work list, and self-service printing system to optimize the whole process of ultrasound diagnosis and treatment so that patients can obtain efficient and high-quality ultrasound medical services in time.

The rest of this article is organized as follows. In the “Materials and methods” section, we describe in detail the demand analysis, the architecture of the system and the design of the various functional modules. In the “Results” section, we compare the data before and after the introduction of the appointment system, including the proportion of appointment channels, appointment time, quality and efficiency of ultrasound examination, etc. In the Discussion section, we discuss the results in terms of accelerating patient access to reliable ultrasound, increasing patient participation, optimizing the diagnosis and treatment environment, and protecting patient privacy. In the last section, we present our conclusions and outline the future work of the system.

## Materials and methods

### Analysis of demands

The purpose of this study was to improve patient satisfaction and sense of medical gain by allowing patients to travel less and information to travel more and to improve the efficiency of the ultrasound department through the dynamic allocation of ultrasound appointment resources (26). Our building objectives include the following:

1) Communication and dynamic management of ultrasound resources. Unified and comprehensive management of resources in each examination room, real-time updates, and subdivisions of resource allocation are achieved.
2) Multiple appointment channels. We made full use of network information technology to realize ultrasound appointments through various channels, such as manual appointment windows, patient self-service device terminals, mobile phone applications and WeChat public accounts.
3) Intelligent appointment rules. Flexible allocation of resources in the examination room, establishment of mutually exclusive rules for inspection items, management of medical order appointment rules, and automatic recommendation of appointment plans.
4) Automatic registration triage call, visual waiting screen, secondary triage. Patients should be triaged to ensure the treatment environment.

Most of our needs come from patients and hospitals. For patients, the main demands are as follows:

1) Shorten the waiting time for medical treatment, including the waiting time for appointment, the waiting time for examination, etc.
2) Medical costs, travel times and unnecessary accommodation costs are reduced.
3) Active participation in the examination, including self-appointment of examination time, being informed of matters needing attention in advance, self-registration for examination on time, checking progress in time, self-printing, etc., is needed.

For hospitals, the main needs are as follows:

1) Efficient resource utilization, real-time resource update, unified allocation.
2) The government should allocate medical resources rationally, improve equipment utilization, and reduce labor costs.
3) To develop a patient-centered diagnosis and treatment process and improve the quality and efficiency of diagnosis and treatment.

### System architecture

**Figure 1:**
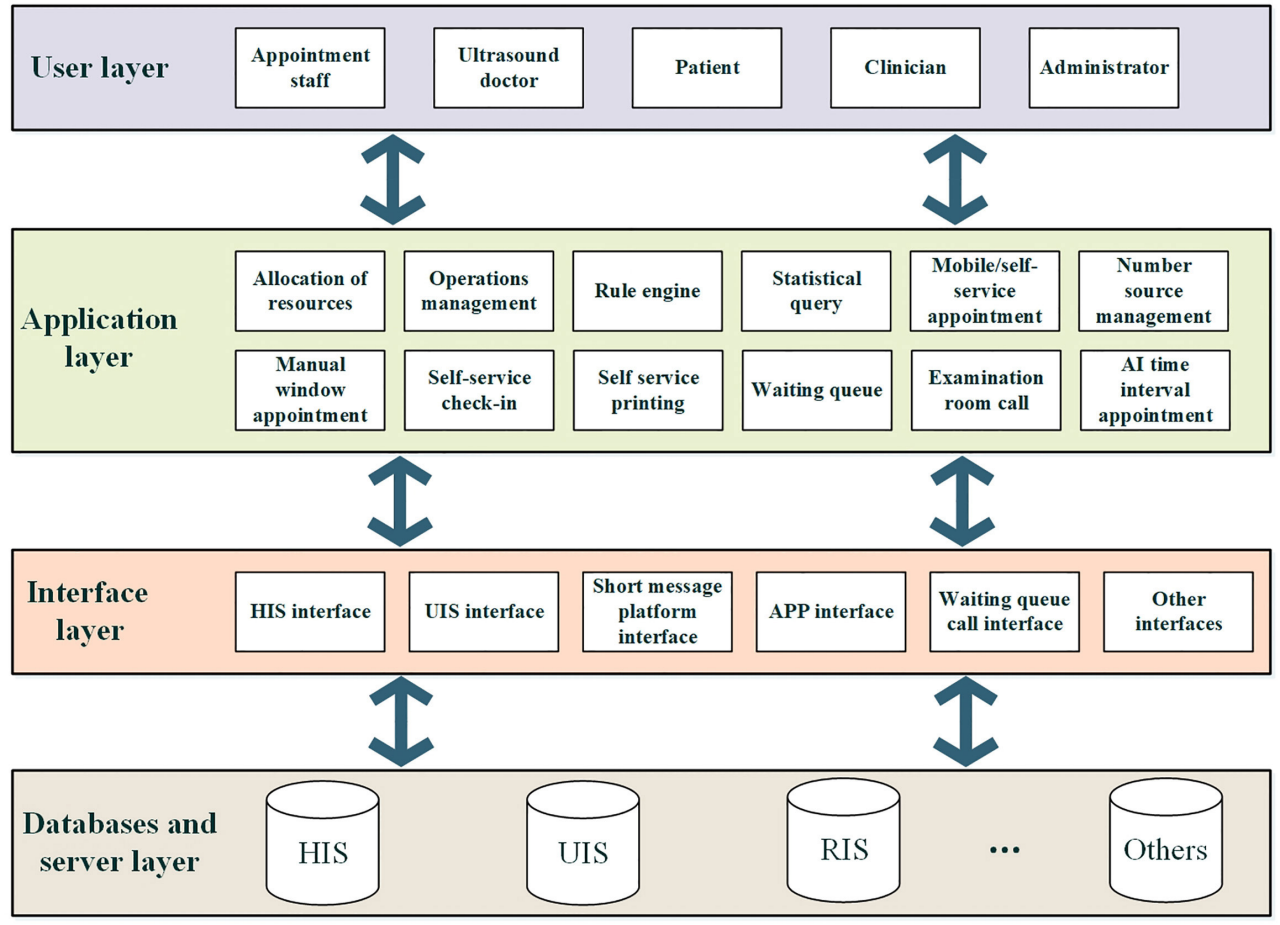
Appointment system architecture diagram.

Through the detailed demand analysis of the system, we designed the overall architecture of the ultrasound appointment system, as shown in Figure 1, which includes mainly the databases and server layers, interface layers, application layers and user layers.

The main functions of each module are as follows:

Databases and server layer: This layer includes all the databases and servers related to the ultrasound appointment system, including obtaining ultrasound order information from the HIS and pushing the appointment list to the ultrasound information system (UIS). The ultrasound appointment system also obtains other examination appointment information from other information systems to avoid examination conflicts. The data of the appointment system are stored in the database and synchronized with the waiting call system for data and status.

Interface layer: The interface layer includes all the database and server interfaces that need to interact with the ultrasound appointment system, including the HIS, UIS, SMS platform, mobile app, waiting and calling system, etc.

The application layer includes the application hardware and software related to the ultrasound appointment system, which can perform medical resource allocation, operation management, appointment rule configuration, manual intelligent time-division appointment, appointment statistical analysis, number source management, self-service appointment system, self-service check-in, self-printing, triage waiting list, and examination room number calling system on the appointment system.

User layer: The users of the ultrasound appointment system mainly include appointment staff, ultrasound doctors, patients, clinicians, and administrators.

### Interface design

**Figure 2:**
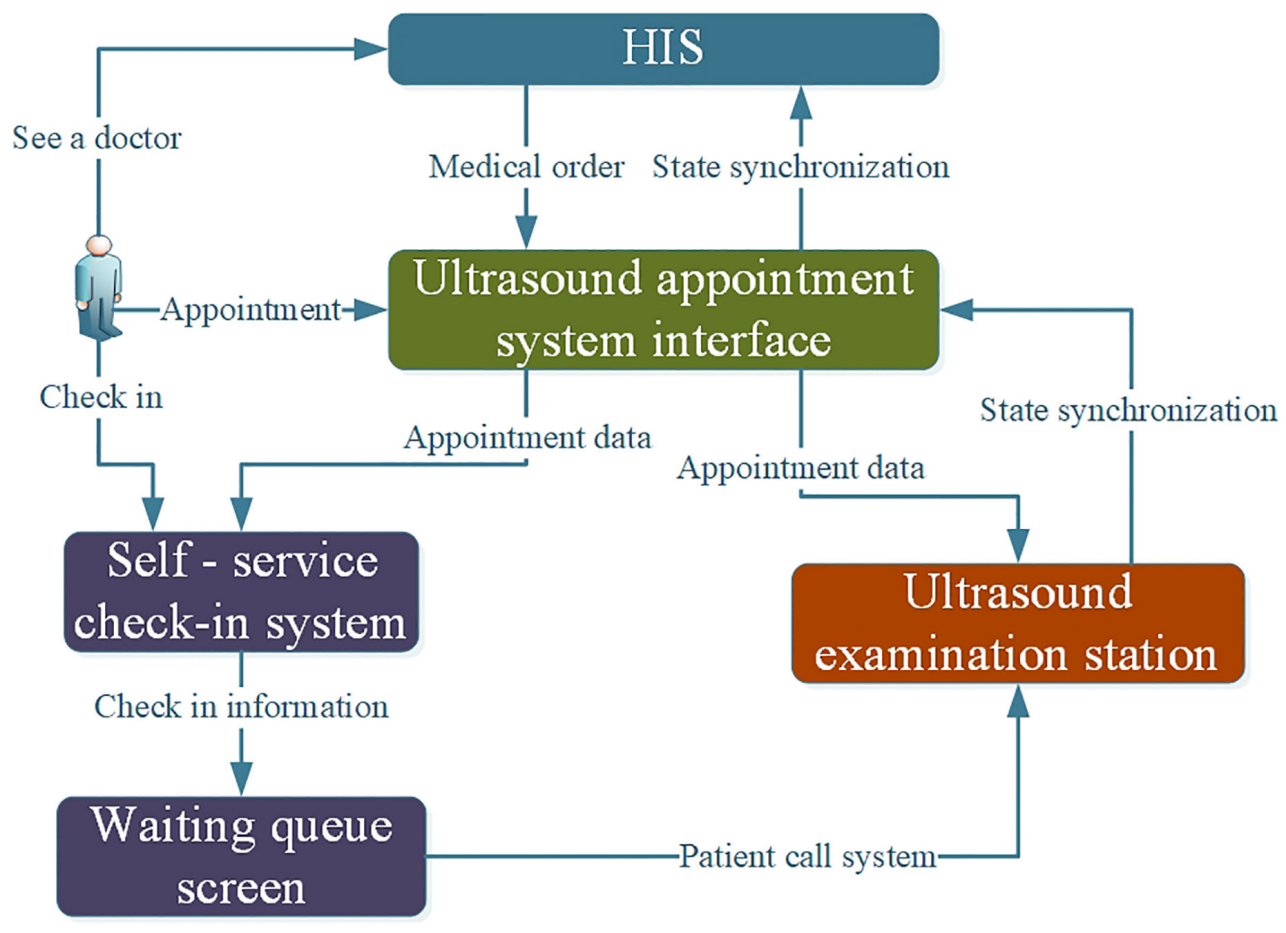
Appointment system interface design diagram.

With the rapid development of hospital informatization and intelligence, hospitals require all kinds of systems to realize interconnections to avoid the formation of information islands; thus, interface design is important in the design of ultrasound appointment systems (27). An HIS is an information management system covering all the business and the whole process of the hospital, and it is the main line of information construction of the hospital. The ultrasound appointment system needs to conduct data interaction with the hospital's HIS system to obtain the patient's ultrasound medical order from the HIS system. The main information includes patient ID, basic patient information, medical order items, prescribing individuals, billing departments, clinical diagnoses, cost information, etc. The ultrasound appointment system also needs to conduct interface data interactions with the ultrasound information system, which mainly includes synchronizing the appointment information to the ultrasound examination workstation and retrieving state synchronization information. The interface design of the appointment system is shown in Figure 2.

### Appointment workflow design

**Figure 3:**
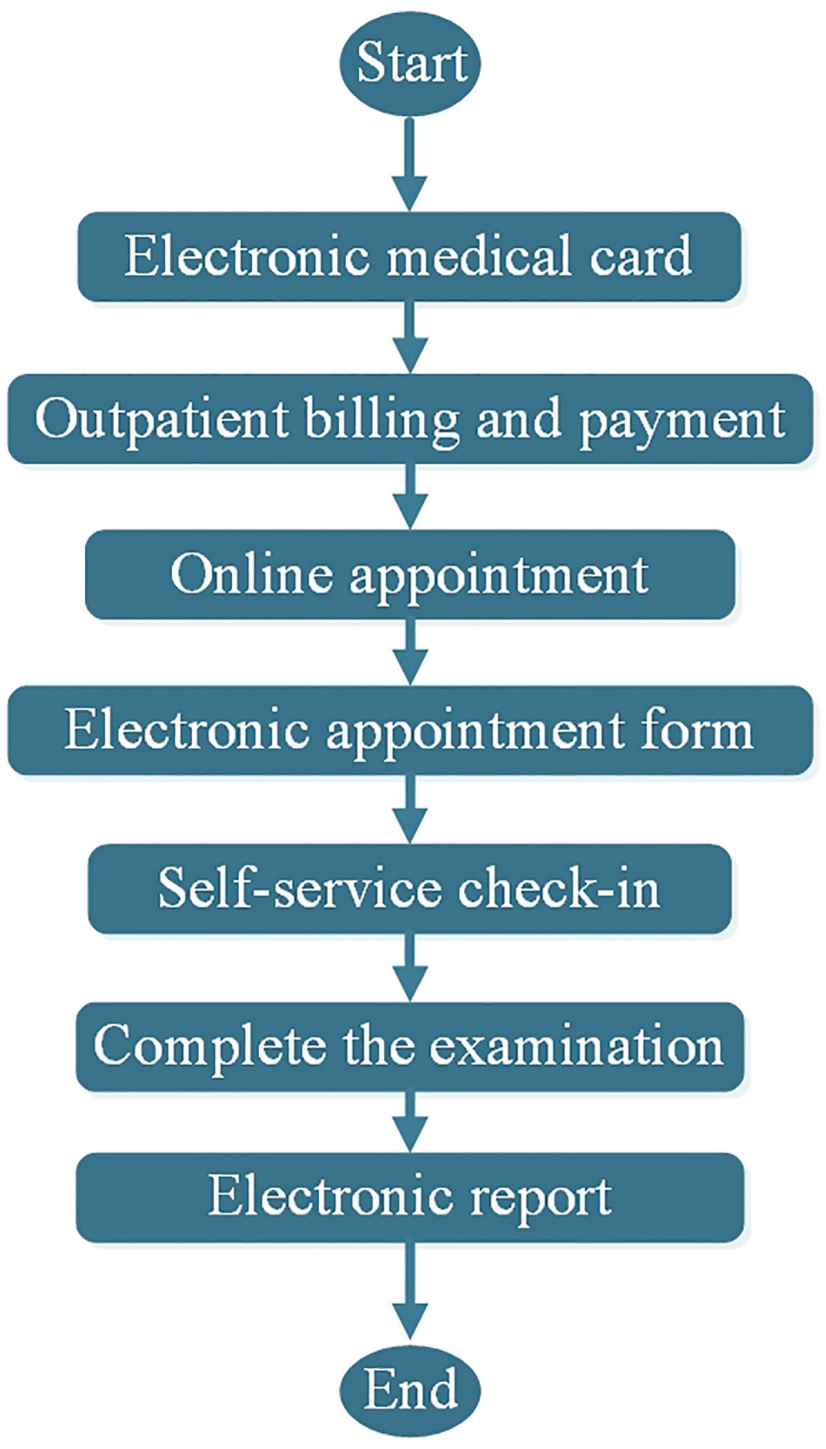
Flow chart related to the ultrasound appointment system.

The ultrasound appointment system in this study adheres to the patient-centered concept. Through mobile apps and self-service terminals, patients are allowed to participate in appointments, registrations and examinations as much as possible, which makes the appointment- and examination-related processes simpler and more friendly. The main workflow is shown in Figure 3.

After completing the registration of the electronic medical card, patients can complete the application and pay for the ultrasound doctor's advice online or through self-billing. Then, they can choose the time to complete the online appointment and obtain the electronic ultrasound appointment form. According to the date, time and place on the electronic appointment form, they arrive at the examination area, complete the registration and wait for the examination call at the self-service terminal. Finally, the ultrasound examination was completed to obtain an electronic report, or a paper report could be printed through the self-service terminal.

### Patient-centered design

**Figure 4:**
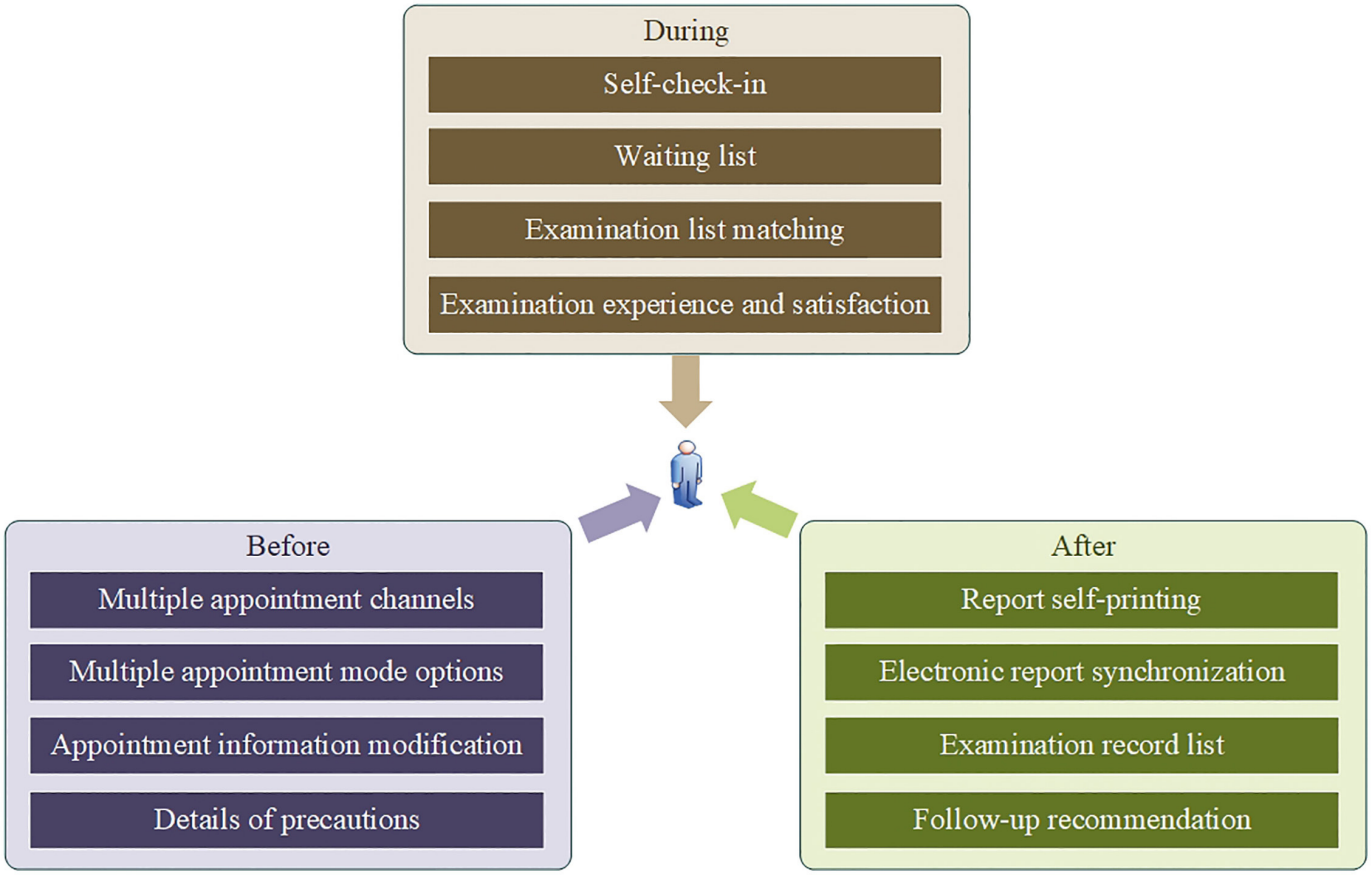
Design of a patient-centered ultrasound appointment system before, during and after ultrasound examination.

With the deepening of medical reform, patient-centered approaches have become increasingly popular, which is also the core idea of this study (28). Let patients run less, information run more, and improved patient satisfaction and sense of access to medical treatment are the core goals of this study. As shown in Figure 4, the patient-centered ultrasound appointment system was carefully designed, and data were collected throughout the entire process before, during and after ultrasound examination. Before ultrasound examination, patients can make an ultrasound medical order through an online outpatient service or self-billing function and then make an appointment through multiple appointment channels, such as mobile phone applications and WeChat public accounts. At the same time, the appointment system provides patients with multiple appointment mode options, including the shortest time, the least round trip, and self-selected time. The appointment data of the appointment system are updated synchronously in real time, and patients can modify the appointment through the appointment system, but the modification is limited; for example, the modification should be completed 1 day before the appointment time to avoid wasting medical resources caused by temporary cancellation of the appointment. When the patient completes the appointment, the appointment system provides the patient with detailed precautions and explanations to ensure that the patient can be prepared for the examination in advance. These designs can reduce the time and cost of patients traveling to and from the hospital and improve the efficiency of appointments. During the ultrasound examination, the ultrasound appointment system pushes the patient's appointment information of the current time period of the day to the triage registration system and the work list of the ultrasound examination room, and the patient first arrives at the waiting area according to the appointment time and location. Upon completion of the patient check-in, the patient's name is displayed in the waiting queue for the examination room call. After the patient enters the examination room, the examination list can be automatically matched by scanning the appointment information. These designs increase patient participation in the ultrasound process, reduce wait time, and reduce patient and information mismatch. After the ultrasound examination, the patient can visit the self-service printer to print the super report through the appointment information of the appointment system. Moreover, the ultrasound results will also be synchronized to the patient's mobile application, forming a list of examination records and providing appropriate follow-up suggestions for the patient. These designs can effectively protect patient privacy, reduce waiting times for printed reports, and effectively organize patient test results to improve patient satisfaction.

### Appointment system management and real-time dynamic resource allocation strategy

#### Authority management

As shown in Figure 1, the ultrasound appointment system faces different users, and different users are configured with different user permissions. For example, patients only have the right to make an appointment and view their own ultrasound examination on a mobile phone or self-service terminal, and the ultrasound department appointment staff have the right to make an appointment on a computer terminal and the right to view appointment data. The administrator has all the rights of the system, including log management and rule configuration.

#### Dynamic rule configuration

To improve the flexibility of the system, all the parameter configurations are open and can be flexibly configured. For example, different branches can be configured to meet the ultrasound appointment needs of different branches (29). The information of the ultrasound examination room can be flexibly configured, such as adding, deleting, modifying, and booking the opening days of the examination room. Ultrasound medical order configurations include medical order codes, combination rules and exclusion rules, workload settings, special medical order binding examination rooms, etc. The properties of the ultrasound examination room can be dynamically deployed according to the actual needs of patients (30, 31). For example, when the appointment time of a specialist ultrasound examination is long, the general examination room can be set as a specialist examination room, and the corresponding medical order configuration can be carried out according to needs, which can shorten the appointment time of a specialist ultrasound examination.

#### Real-time resource allocation

**Figure 5:**
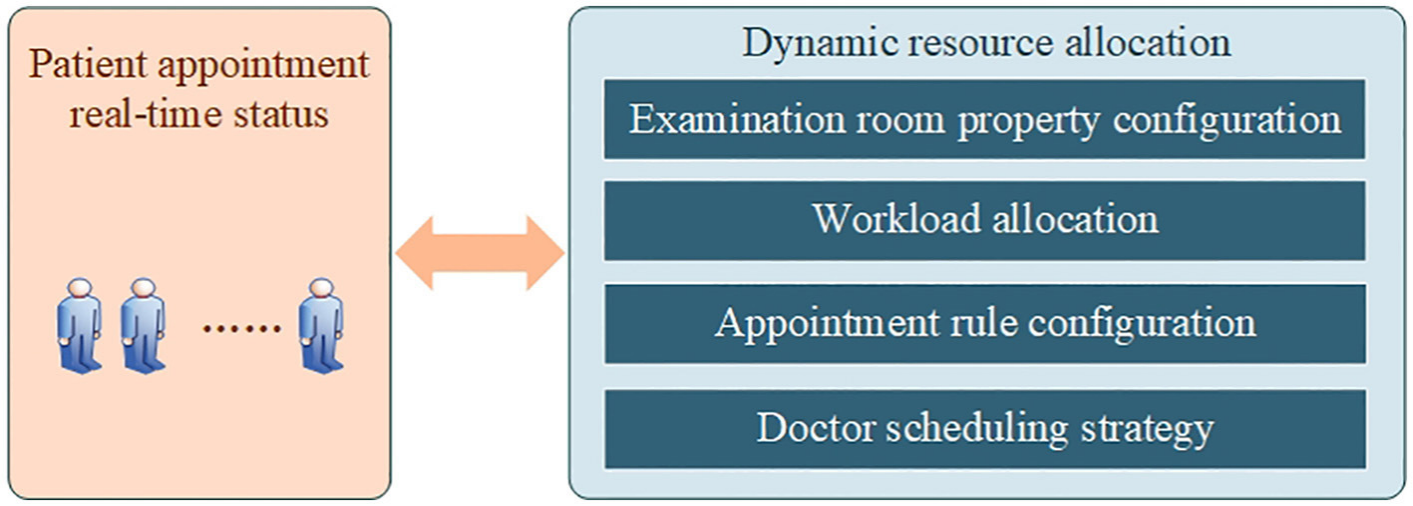
Real-time dynamic resource allocation strategy of the ultrasound appointment system.

Another advantage of the system is the visualized real-time configuration of resources. The ultrasound appointment system can provide real-time and multidimensional data analysis for department managers, including the appointment volume and trend, proportion of different appointment methods, non-attendance rate, resource utilization rate, and appointment workload statistics. These data are presented as visual reports, such as curves and pie charts. The appointment system calculates the position to be booked in each time period according to the set time period and the set number of people and displays it in a visual interface. Different appointment methods, such as self-service appointments and window appointments, are distinguished by different symbols. Administrators and ultrasound appointment staff can also intuitively see the appointment of each examination room, each time period, and position through this interface and can also lock and leave a number for each position in advance. The resources of the examination room are updated in real time, and the appointment information of various channels is summarized to the appointment system and displayed in real time. Administrators can flexibly allocate ultrasound resources according to the appointment situation, as shown in Figure 5. For example, when the number of ultrasound appointments for patients increases, the workload configuration of the examination room can be appropriately increased as needed, or the number of examination rooms can be increased. In contrast, the number of examination rooms should be appropriately reduced. The statistical analysis of multidimensional appointment data will also provide data support for the real-time resource allocation of departments. This helps to realize the rational allocation and efficient utilization of ultrasound medical resources and improve the quality and efficiency of patient ultrasound examinations.

### Online appointment

Through the mobile internet, the online appointment function was designed for patients (32). Patients can complete online ultrasound examination appointments through the mobile app and enjoy a more convenient and efficient appointment experience. After the patient completes the payment of the doctor's order for ultrasound examination, the system will push the information of the ultrasound examination appointment to be made. After the appointment interface is entered, there are generally two options: an intelligent appointment and a self-selected appointment. If an intelligent appointment is chosen, the system automatically obtains the doctor's order of all the ultrasound examination appointments to be made and recommends the most recent appointment plan for the patient according to the set appointment rules and other appointment information of the patient. If you do not like the recommended appointment plan, you can choose the method of self-selected appointment, and the system will list all the appointment dates that can be selected for the patient. The ultrasound appointment system integrates the rules of all ultrasound examinations. For example, abdominal color Doppler ultrasound can only be booked until the morning, and contrast-enhanced ultrasound or intervention can only be booked in the corresponding contrast-enhanced specialist examination room and intervention specialist examination room. There are not only internal appointment rules for ultrasound but also possible interactions between ultrasound and other examinations. For example, abdominal ultrasound usually takes 3 days to perform after gastrointestinal endoscopy or enhanced CT. The ultrasound appointment system can automatically obtain the patient's other examination information through the HIS, which can automatically avoid examination conflicts and cause unnecessary waste of time. At the same time, to further shorten the waiting time of patients for examination, we also adopt the subperiod appointment method, which subdivides half a day into different time periods, usually half an hour (33, 34). In accordance with the order of patients' appointments, different arrival time periods are suggested on patients' electronic appointment forms. In addition to the appointment period, the electronic appointment form displays the full information of the appointment examination room, appointment number, and precautions. The precautions are based on the patient's different ultrasound advice, with different precautions, including fasting, bladder filling, etc., to ensure that the examination can be carried out smoothly.

### Appointment record traceability mechanism

The appointment system in this study has a perfect record traceability mechanism, including the appointment method, executor, IP address, and operation time of each appointment record, which are stored in the database. This is of practical importance for the management of the appointment system, appointment workload statistics, problem traceability, etc. In addition, ultrasound departments often encounter situations in which patients need temporary plus sign examinations. To avoid medical risk caused by the plus sign, this study standardizes the plus sign mechanism to support a reasonable plus sign and avoid reselling the plus sign. Usually, the application is initiated by a clinical doctor with a plus sign, indicating the applicant, the application department, and the reason for the application, and the application is sent to the ultrasound doctor. If consent is obtained, the ultrasound appointment staff performs the plus sign registration and sends it to the ultrasound doctor's examination room, and all the application information is also bound to this examination order for subsequent tracking.

### Self-service check-in and waiting module design

Patient satisfaction is closely related to patient participation in the treatment process and the openness and transparency of waiting. Therefore, this study designed a self-service check-in and printing system to improve patient participation and designed a visual waiting system and voice call system to improve the openness and transparency of waiting. The appointment data are synchronized with the self-service check-in system. After patients arrive at the ultrasound department according to the appointment date and time period on the electronic appointment sheet, they scan the QR code to check and register on the self-service machine. All the registered information is displayed on a large screen in the waiting area, and patients can intuitively see their queuing position. Patients who do not arrive at the examination area in time after calling will be treated as over-registered and will be reregistered. This not only allows patients to clearly see the fairness of the queue but also ensures the smooth flow of the ultrasound examination and a quiet examination environment. After completion of the examination, the ultrasound report is synchronized to the mobile app, and the patient can also pick up the paper ultrasound report from the self-service machine with the examination QR code.

## Results

### Comparison of appointment methods

Before the establishment of the appointment system, window appointments accounted for almost 100% of all appointments. After the appointment system is established, the mobile terminal online appointment function and the hospital multifunction terminal appointment function are added, which greatly reduces the pressure of manual window appointment. However, the manual appointment window has not been completely replaced. One reason is that some people do not have smartphones or do not know how to use them, so they need a manual window to help them complete the appointment. Another reason is that some ultrasound appointments can be made only after the review of window staff, such as ultrasound intervention. We calculated the data 1 month after the establishment of the appointment system, as shown in Table 1. The proportion of manual window appointments decreased from 100% to 23.72%, and with the continuous improvement in appointment rules and the increase in patients' self-appointment awareness rate, this proportion still decreased.

**Table 1:** Comparison of appointment methods.

**Ultrasound appointment channels**	**Appointment number**	**Proportion (%)**
Mobile terminal appointment	79,827	81.42
Self-service terminal appointment	7,635	7.79
Manual window appointment	10,583	10.79

### Comparison of appointment time

The length of the appointment time between the completion of the ultrasound appointment and the execution of the ultrasound examination is one of the key indicators we pay attention to, which is related to whether patients can undergo ultrasound examination in a timely manner to provide ultrasound image data for the clinic and then obtain timely diagnosis and treatment. Ultrasound examinations are usually divided into general ultrasound examinations and specialist ultrasound examinations, and the average waiting time (days) 1 month before and after the introduction of the appointment system is reported in Table 2. Before the introduction of the appointment system, the average number of appointment days for general ultrasound examination and specialized ultrasound examination was 8.6 and 12.3 days, but it decreased to 0.8 and 2.6 days after the introduction of the appointment system, with decreases of 90.7% and 78.86%, respectively.

**Table 2:** Comparison of appointment time before and after the introduction of the appointment system.

**Type**	**Before (days)**	**After (days)**	**Decline (days)**	**Decline (%)**
General examination	8.6	0.8	7.8	90.7
Specialist examination	12.3	2.6	9.8	78.86

### Comparison of ultrasound quality and efficiency

For hospitals and departments, in addition to shortening the appointment time of patients, quality and efficiency are also indicators that need to be focused on, including the quality assurance and efficiency improvement of ultrasound appointments, ultrasound waiting and examination execution. We compared the relevant indicators before and after the introduction of the ultrasound appointment system. As shown in Table 3, the number of window appointment staff was reduced from 5 to 1, the average waiting time for patients (from the time they arrived at the waiting area to the start of ultrasound examination) was reduced from 42 to 11 min, and the number of ultrasound examinations in a month increased from 87,931 to 98,045. The number of error reports decreased from 28 to 9.

**Table 3:** Comparison of ultrasound quality and efficiency.

**Items**	**Before**	**After**
Appointments staff	5	1
Waiting time (min)	42	11
Amount of examination	87,931	98,045
Error reports	28	9

## Discussion

Patient-centered design is the original intention of this system design so that patients can obtain reliable and effective medical services as soon as possible. The patient-centered real-time dynamic resource allocation strategy is multifaceted in optimizing the ultrasound appointment system.

### Accelerate the appointment and examination process to ensure timely access to high-quality and reliable ultrasound medical services for patients

This study proposes a patient-centered real-time dynamic resource allocation strategy to optimize ultrasound appointment systems. This strategy dynamically allocates examination rooms and medical resources by analyzing patients' ultrasound appointments in real time and improves the effectiveness of appointments by setting mutually exclusive rules and doctor's order appointment rules. In addition to providing multiple appointment channels, multiple appointment modes are set for patients to flexibly choose according to their actual needs. These measures significantly shorten the appointment time of patients for ultrasound examinations. As shown in Table 2, the average waiting days for general ultrasound examination and specialist ultrasound examination decreased by 90.7% and 78.86%, respectively. This significant reduction in wait time ensures that patients can access ultrasounds in a more timely manner, thereby improving the efficiency and reliability of healthcare delivery. In addition, the appointment system further reduces the inconvenience caused by examination conflicts by automatically assigning the optimal appointment scheme and limiting mutually exclusive rules. For example, the system can automatically avoid time conflicts between abdominal ultrasound and examinations such as gastroenteroscopy or enhanced CT. The docking of the ultrasound appointment system with the triage calling system and examination room work list also improves the fluency of ultrasound examination, and sonographers can focus more on the ultrasound examination and diagnosis of patients, improving the efficiency of ultrasound examination. As shown in Table 3, owing to the detailed data interface design between the ultrasound appointment system and each system, the number of ultrasound examinations increased by 11.5%, whereas the number of error reports also decreased significantly, which fully indicates that the introduction of the appointment system has improved the quality and efficiency of ultrasound examinations and that patients have obtained more reliable ultrasound medical services.

### Enhancing patient participation and satisfaction

Allowing patients to participate in the whole process of ultrasound examination is one of the measures used to improve patient satisfaction (35). The design of this ultrasound appointment system focuses on the sense of patient participation. Through various channels, such as mobile apps and self-service terminals, patients can choose and adjust their appointment time independently, which enhances the initiative and convenience of patients. According to their needs and actual situation, patients can choose a variety of appointment modes, such as the shortest time, the least number of round trips or the optional time, which improves patient satisfaction. After the appointment is completed, the appointment system prompts you to prepare for the ultrasound examination. After patients arrive at the examination area, through the self-service check-in system and visual waiting screen, patients can know their own examination progress and waiting situation in real time, improve the transparency of the queuing system, and further enhance the sense of participation and experience of patients. After the examination is completed, the patient will print the ultrasound report as needed, while the ultrasound results will be synchronized to the patient's mobile app and provide the patient with appropriate explanations and follow-up recommendations. These user-friendly designs of the ultrasound appointment system allow patients to fully participate in the whole process of ultrasound examination while respecting patients' independent choices, thus improving patient satisfaction.

### Optimization of the treatment environment and improvement of the order

The effective triage of patients is an important measure for building an optimized treatment environment. As shown in Table 1, the proportion of online appointments is large, and patients can make ultrasound doctor orders and ultrasound examination appointments online, which can greatly reduce the number of patients returning to the hospital, improve the effectiveness of patients arriving at the hospital, and reduce the flow of patients in the hospital. The hospital treatment environment should be optimized. Moreover, the ultrasound appointment system calculates the patient's expected examination time according to the patient's appointment sequence number through statistical analysis of the average examination time of each doctor's order, and the patient can reach the waiting area according to the prompt time of the ultrasound appointment sheet. As shown in Table 3, the time from the arrival of patients in the waiting area to the start of the ultrasound examination was reduced from 42 to 11 min, which greatly reduced the number of patients in the waiting area and ensured a relatively quiet examination environment for the ultrasound doctors. Furthermore, by visualizing the waiting screen and the secondary triage system, patients can see the queue clearly and transparently, avoiding conflicts caused by unclear queues. These measures are very effective in optimizing and improving the environment and order of medical treatment.

### Privacy protection and humanistic care

Patient privacy protection and humanistic care are also the focus of this ultrasound appointment system. The appointment system ensures the security and privacy of patient information through permission management to avoid the risk of information disclosure. The system provides a variety of appointment modes and detailed examination precautions so that patients can choose and prepare according to their actual situation and needs, reflecting attention to and respect for the individual needs of patients. In addition, various designs that allow patients to participate in the whole process of ultrasound examination, including dynamic adjustment of ultrasound resources to shorten the appointment time, self-service registration, and self-service printing, also reflect the emphasis on humanistic care.

### Comparison of different appointment strategies

At present, there are a variety of studies on appointment strategies for ultrasound and other medical examinations at home and abroad, including independent appointments, overbooking, classified appointments, free ride appointments by carpools, intelligent appointments, etc. Independent appointments are among the easiest and most commonly used strategies to implement. In this appointment strategy, the appointment of the ultrasound department is relatively independent from that of the radiology department and other medical examination departments, which is conducive to the internal resource allocation of the ultrasound department, simplifying the appointment process, and improving the appointment efficiency (36–38). The greatest drawback of the ultrasound department is that it cannot be combined with other medical examinations, which usually leads to patients returning to the hospital multiple times to complete different examinations. Sometimes there are even conflicts between test items that cause invalid appointments, thus extending the overall time of the patient's visit. Overbooking is another common appointment strategy, which involves making more appointments on the basis of the original appointment workload (39, 40). This strategy is used to avoid the waste of medical resources caused by some patients missing appointments. However, its disadvantages are also obvious because the absence of patients is difficult to predict; when patients do not miss appointments, the workload of doctors increases, and the quality of ultrasound examination is difficult to guarantee. The classification of patient appointments is another appointment strategy. Under this strategy, patients are divided into emergency patients and routine patients to ensure that patients in urgent need of treatment can have priority access to ultrasound examination resources (13). However, the classification standard is controversial to some extent; sometimes, there are cases of fake emergency treatment, and the appointment of routine patients has basically not improved. A small number of studies have examined ride-sharing services or offering free rides to reduce missed appointments, a strategy that can be effective for a small number of patients with transportation difficulties and can improve the immediacy of appointments but does not significantly reduce missed appointments (41, 42). An intelligent appointment system can analyze historical data to optimize the allocation and utilization of medical resources, but it has several problems, such as high technical dependence, potential data security risks, and poor generalizability of the model (43). The advantages and disadvantages of different current appointment strategies are shown in Table 4.

**Table 4:** Advantages and disadvantages of different current appointment strategies.

**Strategy**	**Advantages**	**Disadvantages**
Independent appointment	Simplify the ultrasound appointment process and improve the appointment efficiency	Inability to coordinate with other medical tests leads to multiple trips to the hospital for different examinations
Overbooking	Avoid the waste of medical resources caused by some patients missing appointments	Increase the workload of the doctor and the quality of the examination is difficult to guarantee
Classified appointment	Ensure emergency patients have priority access to ultrasound	The classification criteria were controversial and did not improve routine patient appointments
Carpool appointment	Improve the timeliness of appointment for a small number of patients with transportation difficulties	Implementation was difficult, costly and failed to reduce missed appointments
Intelligent appointment	Optimize the allocation and utilization of medical resources	High technology dependence, potential data security risks, model generalization

The patient-centered real-time dynamic resource allocation strategy in this study combines the advantages of multiple appointment strategies while minimizing their disadvantages. This study is expected to provide a positive reference for existing research on ultrasound and other medical appointment systems. This strategy has many advantages, through the establishment of a unified database with other medical examinations and a mutually exclusive medical order association system, the overall appointment efficiency of patients can be improved while reducing the transportation cost and time cost associated with patients returning to the hospital multiple times. Through the design of online and offline combinations of multichannel and multimode appointments, the appointment process can be simplified. Through the combination of outpatient “people may take breaks, but the machines continue running” and emergency and hospitalization service modes, doctors' shifts can be flexibly adjusted according to patients' appointment conditions in the limited ultrasound examination space, which can improve the efficiency of equipment use and effectively shorten the appointment time of patients. Through machine learning analysis and deep mining of historical appointment data, the time required for various ultrasound examinations can be budgeted, and the estimated examination time of patients can be calculated according to the order of patient appointments. The system makes time-sharing appointments for patients, thus further shortening the time for patients to wait for examinations, ensuring a good medical environment and avoiding medical chaos. Through real-time statistical analysis, the missed appointment data can be supplemented in time to make full use of valuable medical resources and avoid overbooking. By combining the ultrasound appointment system with the self-service registration system, waiting system and self-service printing system, the participation and satisfaction of patients can be improved, and the spirit of humanistic care can be reflected while protecting the privacy of patients. By designing a visualization system to display appointment data in real time and combining information technology with data mining technology, the system's ease of use, interpretation, traceability and generalizability can be improved.

## Conclusion

The application of a patient-centered real-time dynamic resource allocation strategy in an ultrasound appointment system is introduced in this work. This study comprehensively analyzed patients' demand for ultrasound appointments; designed the system architecture, function modules, data interfaces and workflows on the basis of patients' demand; and integrated online and offline data, diversified appointment modes, automated medical order attributes and mutually exclusive rules, visualized appointment data, and integrated data between systems. The real-time appointment data of patients are used to make dynamic allocations of resources to the ultrasound appointment system. Through comparative analysis of the data before and after the introduction of the appointment system, the system effectively accelerated the process of patient appointment and examination, shortened the time of ultrasound appointment and improved the quality and efficiency of ultrasound examination. The ultrasound appointment system under this strategy can enhance the participation of patients in the whole process of ultrasound appointment and examination and improve the hospital treatment environment and order. Patient privacy is effectively protected, and patient satisfaction is greatly improved. These patient-centered designs reflect the hospital's emphasis on humanistic care and patient-centered philosophy, while also improving the hospital's ultrasound medical service performance in extreme disaster events and disaster preparedness for various emergency situations. In the next step, high-tech technologies such as artificial intelligence and data mining will be gradually introduced to continuously innovate and optimize ultrasound appointment and diagnosis and treatment models to better serve patients.

## Data Availability

The raw data supporting the conclusions of this article will be made available by the authors, without undue reservation.
